# Supplementary material for: The ARMADILLO text message intervention to improve the sexual and reproductive health knowledge of adolescents in Peru: Results of a randomized controlled trial
Source: PLoS One. 2022 Feb 10;17(2):e0262986. doi: 10.1371/journal.pone.0262986 (PMC8830715; doi:10.1371/journal.pone.0262986)
Supplement: Attachment — Submitted filename: ResponsetoReviewers 06.24.2021.docx [file pone.0262986.s003.docx]

Additional Editor Comments:

After careful review, although both Reviewers identified potential strengths of the highlighted intervention and its potential impact on adolescent health, they also identified a number of weaknesses. Taken collectively, in order to consider this publication further, it will need a major revision addressing each of the reviewers noted general critiques regarding the presentation of the results and in presenting the implications of the findings addressing the effectiveness of the intervention on the sexual and reproductive health knowledge of Peruvian adolescents.

*Our sincere thanks to the editor and the two reviewers for taking time to provide thoughtful feedback during what has been an extremely difficult time. We feel the manuscript is greatly strengthened as a result of the changes suggested. Our responses can be found in line below, and in tracked changes in the manuscript.*

Journal Requirements:

When submitting your revision, we need you to address these additional requirements.

1. Please ensure that your manuscript meets PLOS ONE's style requirements, including those for file naming. The PLOS ONE style templates can be found at

<https://journals.plos.org/plosone/s/file?id=wjVg/PLOSOne_formatting_sample_main_body.pdf> and

<https://journals.plos.org/plosone/s/file?id=ba62/PLOSOne_formatting_sample_title_authors_affiliations.pdf>

*This is now done.*

2. We note that you have indicated that data from this study are available upon request. PLOS only allows data to be available upon request if there are legal or ethical restrictions on sharing data publicly. For information on unacceptable data access restrictions, please see <http://journals.plos.org/plosone/s/data-availability#loc-unacceptable-data-access-restrictions>.

In your revised cover letter, please address the following prompts:

 a) If there are ethical or legal restrictions on sharing a de-identified data set, please explain them in detail (e.g., data contain potentially identifying or sensitive patient information) and who has imposed them (e.g., an ethics committee). Please also provide contact information for a data access committee, ethics committee, or other institutional body to which data requests may be sent.

*Our protocol was designed in 2016 and at this time, no provisions were required with regard to data availability and public sharing. As such, these were never included in the protocol and we never received ethics clearance to publish data, de-identified or otherwise. For results published from the Kenya site under the same global study (therefore, facing the same issue), our data availability statement has included contact information for the relevant IRB board. We have proposed similar language in the revised manuscript’s data availability statement which now reads:*

*“Data cannot be shared publicly as consent procedures for participants and the trial protocol did not include making survey results publicly available. Data are securely stored on the HRP e-Archive system, part of World Health Organization’s (WHO) official Enterprise Content Management (ECM) platform. Deidentified data can be made available on reasonable request to the corresponding author, following approval from the Comité Institucional de Ética en Investigación at the Universidad Peruana Cayetano Heredia (contact* [*duict.cieh@oficinas-upch.pe)*](mailto:duict.cieh@oficinas-upch.pe))*.”*

*We hope the editor will consider accepting this as a compromise.*

b) If there are no restrictions, please upload the minimal anonymized data set necessary to replicate your study findings as either Supporting Information files or to a stable, public repository and provide us with the relevant URLs, DOIs, or accession numbers. Please see <http://www.bmj.com/content/340/bmj.c181.long> for guidelines on how to de-identify and prepare clinical data for publication. For a list of acceptable repositories, please see [http://journals.plos.org/plosone/s/data-a	vailability#loc-recommended-repositories](http://journals.plos.org/plosone/s/data-availability#loc-recommended-repositories).

 We will update your Data Availability statement on your behalf to reflect the information you provide.

 3. Please include captions for your Supporting Information files at the end of your manuscript, and update any in-text citations to match accordingly. Please see our Supporting Information guidelines for more information: [http://journals.plos.org/plosone/s/supporting-information](about:blank).

Reviewer #1: Abstract
- it is more typical to include a brief background of the study in addition to the study objective (rather than the objective alone) in the 'background' section of the abstract.
*We appreciate this comment; the brief background is a result of trying to be respectful of the journal’s abstract word limit, while also fulfilling CONSORT abstract reporting requirements. We are happy to add more detail if the editor allows; however given we are already 56 words over limit, we hope the reviewer will accept the brief background section as is.*

Background
- in the first paragraph you probably mean "In Peru, the rates of adolescent pregnancy has remained unchanged over time..."

*Thank you for catching this. We have made the change as suggested.*

Methods
- more information should be provided about how the adolescents were selected; it is not sufficiently clear to say "randomly selected male and female adolescents who met the eligibility criteria".

*Thank you for this - we have now included additional information about the selection of adolescents for study participation, under Methods – Participants – Recruitment and baseline assessment.*

- please check the parameters cited in your sample size calculation. A quick check (e.g. using the calculator at <https://www.sealedenvelope.com/power/binary-superiority/>) shows that 373 participants per arm i.e. 746 participants in the main treatment and control arm combined, not counting the third arm, are required to demonstrate a change in outcome from 55% to 65% (10% absolute change) with 80% power at the 5% level of significance; and this is before inflating the sample for expected dropouts. If a different approach has been used for this calculation, e.g. one based on the mean number of correct responses, this should be described more clearly.

*To calculate the sample size, our calculations are based on the mean number of myths believed. For the mean number of myths, we treated the number as a continuous variable and used a baseline level of 0.55. We made a few changes in the manuscript to better present this: “based on the assumptions that the mean baseline level of belief in myths index is 0.55.”*

*A mean value of 0.55, a change in outcome of 0.10, and a standard deviation of 0.30, with 80% power at the 5% level of significance, result in a sample size of 188 per arm.*

*For the loss to follow-up (LFU), we used the n/(1-LFU%) formula. The related calculation of 188/(1-0.20) results in a sample size of 235 per arm.*

- additionally, there needs to be some justification for the assumption of a baseline outcome of 55% and the 10% improvement, e.g. previous work suggesting this baseline level of the outcome; important effect size, e.t.c.

*For the baseline outcome of 0.55, we reviewed the literature for other studies that had measured similar constructs, specifically, contraception-related myths and misconceptions. Family planning myths indexes are not commonly used, so while literature was extremely limited, we were able to draw from one study which took place in Kenya, Senegal, and Nigeria with individuals from age 15-49, cited in the main study protocol, to inform the global estimate. For Peru, we then confirmed the relevance of these findings using a study of 11-19 year old adolescents in northeastern Brazil. Among other concepts, this latter study measured contraception-related attitudes among participants and also found a baseline value of approximately 55% on the items that are most relevant to the items measured in our study. Both studies are now cited in the sample size section.*

*For the 10% improvement, at the time this study was designed (2015/2016), there was a lack of robust evaluations of similar interventions that could help inform our sample size by showing what change we might anticipate to see. Expectations from informal conversations with colleagues in the digital health space were that such interventions could anticipate a notable increase (20%+ change from baseline to endline, following the multi-week intervention). Using this as a starting point, we opted for a more conservative estimate (10%), wanting to ensure that we would be powered to detect smaller changes, should these expectations turn out to be too aspirational (as they were).*

- an analysis of a change-from-baseline score should normally include adjustment for the baseline score as a covariate in the regression model (see the 'change from baseline analysis section in: Committee for Proprietary Medicinal Products. Points to consider on adjustment for baseline covariates. Stat Med 2004;23(5):701–9. Available from: <http://www.ncbi.nlm.nih.gov/pubmed/14981670>). This is equivalent to modelling the score at endline as the outcome adjusting for baseline score (among other covariates if desired). When you make this change in your analysis please update the abstract to indicate that the outcome was the baseline-adjusted change in the mean score. If the data are in 'long' form (with one variable for the knowledge score and a time indicator for the survey), then a model for the score variable as the outcome with the time variable, group variable (i.e. intervention v. control) and a time-group interaction can be used to estimate baseline-adjusted effects (see doi:10.1016/S0140-6736(18)31782-3 for an example of this and the appendix of doi:10.1186/1471-2431-11-109 for a detailed explanation of this approach, ignoring the adjustments for clustering which are not present in ARMADILLO).

*Thank you for this - For our regression model, the primary outcome is delta or the subject-specific change in the mean score on the “index of myths and misconceptions around contraception” between baseline and endline. We have described this in detail under Methods – Primary outcome. Therefore, each individual’s baseline score is already taken into account in the analyses, since all of the analyses are of the delta or the change in the score from baseline to endline, not of the endline score.*

Results
- please include 95% confidence intervals when you report the effect estimates in the main text, i.e. estimate, 95%CI and p-value. Do not report SDs for outcomes in the text of the results or in table 2, as these are used for descriptive purposes and not for inference. However, you may (indeed, should) report baseline scores in Table 1 with their SDs.

*Thank you for this comment. As requested, we have now the 95% confidence intervals to the p-values for the effect estimates in the main text. We also removed the SDs for the outcome measures at in the text of the results. Finally, we also added the baseline outcome data, including the SDs, into Table 1.*

- Table 2 and Table 3 can and should be improved. They should have the mean scores at endline (with SEs) of each of the three arms in separate columns, and the pairwise unadjusted and adjusted differences with 95%CI and p-values.

*On reading this comment, we reviewed Tables 2 and 3 for opportunities for improvement. Both tables have the mean scores for the three timepoints (baseline, endline, follow-up) in three separate columns and the pairwise unadjusted and adjusted differences, with 95%CI and p-values for each, in additional separate columns.*

Reviewer #2: Thank you for the opportunity to review this manuscript, which describes the results of a rigorously designed study of a SMS to improve sexual and reproductive health knowledge among teens in Lima, Peru. Based on the abstract and the first 14 pages of the submission, I could find little wrong with the study or the paper (though I'd preferred the paper be written in the active voice so that it was clearer who was doing the research rather than it being done by, assumedly, all of the authors at once.)

But on page 14 the authors state as a limitation that of the 236 people randomized into the first of the RCT's three groups "relatively few (28, or 13.4% of participants in the arm)" actually received the full intervention. I'd say that the use of the word "relatively" is a major understatement! Any researcher who claims never to have made a massive blunder in their research at some point is likely a liar, and I think too many of these mistakes never make it to manuscripts, usually because the authors are embarrassed and don't try. So, I appreciate the authors' honesty in including the paragraph about this major mishap on such a big project. But it should not have been relegated to a paragraph about limitations, and this huge problem with the data collection should certainly not have been left out of the abstract, which makes claims about the results of the study and Arm 1 that are extremely suspect with a sample size so small. After the fact, I noticed that the n for the whole study was in the abstract but not the n of each arm.

*Thank you for this comment. We assure the reviewer it was not our intent to bury key intervention delivery issues late in the paper and we have endeavoured based on the reviewer’s comment to bring pertinent information to the fore. We have now included reference to the small sample size now in the abstract, explicitly referenced it twice in the text of the results (in the content exposure section and the per protocol analysis section, whereas before it was only in the latter) and moved it further up in the discussion. The sample size remains visible in the relevant Tables, as before.*

*As to why we keep the other analyses in the paper and continue to present them first: We are somewhat bound by norms in presenting primary outcomes for RCTs in that we needed to present the primary outcome first. Our primary outcome was always an intent-to-treat (ITT), and as with standard practice, was established during protocol development, with specific analysis methods described in detail during statistical analysis plan development (including development of shell tables), all of which took place before any analysis started – all to ensure no bias in how the analysis was approached (e.g. fishing for exciting results).*

*Sample sizes of ITT outcomes, unfortunately for this write-up, are determined by how many participants complete baseline and endline surveys. Given what we discovered about intervention delivery for the intervention arm, we felt that presenting a paper with the ITT analysis alone (again in line with standard practice to present primary outcomes) would omit key information about the realities of intervention delivery. In short, we would see that there was no significant effect in the on-demand arm, but readers would never know that this may be due to a majority of on-demand participants not having received the intervention as intended.*

*For this reason, and in consultation with the statisticians on this paper (the first and seventh authors), we decided to include the content exposure and per protocol analyses, where we could ensure that the ‘real world’ sample size issues could also be presented and provide more potential context for null results.*

In addition, I don't think it's a great idea to claim that one of the study's strengths is the use of per protocol analyses that revealed the Arm 1 intervention failure. It's not a strength that no one notice the error until the data analysis. The researchers in Lima should have been monitoring the data flow in real time, and a nearly 90% difference in participation in one arm would have alerted the researchers that something was wrong and needed to be dealt with immediately. Or maybe something else happened -- but whatever it is, it needs to be explained in more detail than what is given.

*We’ve now added a paragraph clarifying why real-time data monitoring did not happened, which we hope addresses this reviewer’s point. We also tone down the language around per protocol analysis inclusion being a strength. We do however, highlight the importance of digital health interventions looking beyond mere ITT, for the ability to measure effect when an intervention is implemented as intended.*

All this said, I think this paper has potential value simply for the analysis of Arm 2 and Arm 3. But any revision needs to state bluntly and early what the sample size in the arms were and why the discrepancy between Arm 1 and Arms 2 and 3 exists. And since the results of the RCT (with or without Arm 1) are rather unexciting, an examination of what went wrong and the lessons learned would be much more valuable than a formulaic description of a very small, if statistically significant, effect.

*We hope that the reviewer is satisfied with the revision, which (as described above) balances the requirements of RCT reporting while also making clear the important limitation of this study and what went wrong. Given that digital health research is in its own adolescence, we find it important to present our findings (the good and the bad) transparently.*
