# Supplementary material for: The ARMADILLO text message intervention to improve the sexual and reproductive health knowledge of adolescents in Peru: Results of a randomized controlled trial
Source: PLoS One. 2022 Feb 10;17(2):e0262986. doi: 10.1371/journal.pone.0262986 (PMC8830715; doi:10.1371/journal.pone.0262986)
Supplement: Attachment — Submitted filename: ResponsetoReviewers 03.11.2021.docx [file pone.0262986.s004.docx]

Journal Requirements:

Please review your reference list to ensure that it is complete and correct. If you have cited papers that have been retracted, please include the rationale for doing so in the manuscript text, or remove these references and replace them with relevant current references. Any changes to the reference list should be mentioned in the rebuttal letter that accompanies your revised manuscript. If you need to cite a retracted article, indicate the article’s retracted status in the References list and also include a citation and full reference for the retraction notice.

*Noted. We are not aware of any retractions.*

Additional Editor Comments (if provided):

As noted in the reviewers comments, you have been very responsive to prior reviewer comments, resulting in a stronger, clearer manuscript. However, there are a few minor lingering issues noted by reviewers requiring further attention. Specifically, Reviewer 1 has some additional recommendation regarding the presentation of summary statistics in Tables and Reviewer 2 has noted where further clarification regarding 'inclusion criteria' should be considered.

*Our thanks to the two reviewers for the continued interest in the manuscript. We are happy to accept their changes as proposed.*

Review Comments to the Author

Reviewer #1: If the last two rows of Table 1 are showing mean and standard deviation (SD) like the age row at the top, please indicate this as you have done for other continuous variables in the table.

As indicated in the previous round of comments, Table 1 (descriptive results) should normally report means and SDs for continuous variables - you have done this. However inferential results such as have been reported in Table 2 and 3 should report means and standard errors (SE) not SDs for continuous outcomes. The rest of Table 2 and 3 are fine.

*Accepted. Table 1 is now appropriately labelled and Tables 2 and 3 report SEs rather than SDs.*

I am satisfied with the other changes made in response to previous reviews.

*Noted with thanks.*

Reviewer #2: Thank you for the chance to review this revision. The authors did a fantastic job responding to the comments, and I greatly appreciated the detail in the discussion of what went wrong and why. I think this makes the paper more valuable than the data alone. My one suggestion is minor. On page 13 of the revision, the authors state, "The criteria for inclusion in this analysis resulted in a very small sample size for on-demand arm..." and I eventually figured out what they meant, because of the wording I initially thought they were referring to the inclusion criteria for participants (which was discussed just on the previous page, 12) rather than the criteria for inclusion in the ITT data (explained on page 10). I suggest rephrasing the sentence to be clearer on that issue. Otherwise, this paper looks great. Thank you again for the chance to review the original and revision.

*Thank you for the kind words. We agree with the final comment and have rephrased as follows “The criteria for participants to be included in this analysis resulted in a very small sample size for the on-demand arm (n=28), limiting the ability to interpret results from this arm.”*
